# Taxonomically Restricted Genes in *Bacillus* may Form Clusters of Homologs and Can be Traced to a Large Reservoir of Noncoding Sequences

**DOI:** 10.1093/gbe/evad023

**Published:** 2023-02-15

**Authors:** Wojciech M Karlowski, Deepti Varshney, Andrzej Zielezinski

**Affiliations:** Department of Computational Biology, Adam Mickiewicz University in Poznan, Uniwersytetu Poznanskiego 6, Poznan, Poland; Department of Computational Biology, Adam Mickiewicz University in Poznan, Uniwersytetu Poznanskiego 6, Poznan, Poland; Department of Computational Biology, Adam Mickiewicz University in Poznan, Uniwersytetu Poznanskiego 6, Poznan, Poland

**Keywords:** taxonomically restricted genes, orphan genes, gene evolution, bacteria, Bacillus

## Abstract

Taxonomically restricted genes (TRGs) are unique for a defined group of organisms and may act as potential genetic determinants of lineage-specific, biological properties. Here, we explore the TRGs of highly diverse and economically important *Bacillus* bacteria by examining commonly used TRG identification parameters and data sources. We show the significant effects of sequence similarity thresholds, composition, and the size of the reference database in the identification process. Subsequently, we applied stringent TRG search parameters and expanded the identification procedure by incorporating an analysis of noncoding and non-syntenic regions of non-*Bacillus* genomes. A multiplex annotation procedure minimized the number of false-positive TRG predictions and showed nearly one-third of the alleged TRGs could be mapped to genes missed in genome annotations. We traced the putative origin of TRGs by identifying homologous, noncoding genomic regions in non-*Bacillus* species and detected sequence changes that could transform these regions into protein-coding genes. In addition, our analysis indicated that *Bacillus* TRGs represent a specific group of genes mostly showing intermediate sequence properties between genes that are conserved across multiple taxa and nonannotated peptides encoded by open reading frames.

**Significance**

Almost every newly sequenced genome contains genes with no traceable evolutionary history. Such genes may often represent determinants of unique organismal traits. By applying rigorous annotation parameters, we explored the genomic space of *Bacillus* bacteria that encompasses exceptionally diverse species. We found that such genes may form families of restricted taxonomic distribution and can be traced to the noncoding sequences of both closely and distantly related bacteria. In this way, we defined a large reservoir of noncoding sequences that could be transformed into protein-coding genes by a few single sequence substitutions. We show that taxonomically restricted proteins have specific properties when compared with peptides encoded by randomly selected open reading frames and evolutionarily mature annotated proteins.

## Introduction

The identification of genes that do not show similarity to sequences from other organisms has raised understandable interest (Jordan et al. 2001) as they may represent genetic determinants of lineage- or species-specific phenotypic traits. Often, these putative genes were suspected to be annotation artifacts, however, some have been seen as functional components of various genomes (Daubin and Ochman 2004). As data related to gene sequences without detectable homologs has accumulated, the definition and naming of these genes has also changed. Initially, they were defined as “orphans” or “singletons” (previously undiscovered genes in a single species) (Dujon 1996; Domazet-Loso 2003). Then they were considered ORFans (open reading frames with no detectable sequence similarity to any other sequence) (Fischer and Eisenberg 1999). Eventually, they were defined as lineage-specific (Cai et al. 2006) or taxonomically restricted genes (TRGs) (Wilson et al. 2005). The latter term being the most flexible as it allows for the definition of unique genes at various levels of taxonomic classification.

One of the more puzzling questions associated with TRGs concerns their origin. Some of the more obvious features of TRGs suggest that they may represent spurious annotations. In particular, the shorter length of taxonomically restricted sequences in comparison to genuine proteins has raised suspicions of over-annotation of the open reading frames (ORFs), especially when they overlap with regions containing small, noncoding RNAs (Wilson et al. 2007). It has also been proposed that TRGs are products of algorithmic shortcomings of the genome annotation of DNA regions with low GC content (Macalalad et al. 2012). Another reason for the absence of TRG homologs could be related to the coverage and sampling bias of public genomic repositories (Siew and Fischer 2003b) and/or limitations of homology detection algorithms (Vakirlis, Carvunis, et al. 2020; Weisman et al. 2020).

When considering a natural origin of TRGs, one source of new functional sequences would be duplication and divergence (Ohno 2014). In such cases, these proteins would either be distributed among species that current sequence data resources do not include, or they would have evolved rapidly beyond the recognition of available algorithms (Domazet-Loso 2003; Tautz and Domazet-Lošo 2011; Vakirlis, Carvunis, et al. 2020). Some studies have suggested the involvement of horizontal gene transfer (Entwistle et al. 2019) that could impede sequence similarity search when executed in a limited range of reference datasets that may not include the source genome(s). Finally, TRGs may represent novel genes—sequences encoding functional proteins and RNAs that originate from previously noncoding regions of the genome (Domazet-Loso 2003; Carvunis et al. 2012; Neme and Tautz 2013). In this case, similarity searches within the protein space would not result in the identification of homologs. However, it should be possible to identify the corresponding genome fragments in closely related organisms. Although origination via a de novo mechanism could appear unlikely, several studies demonstrated, to various extent, the potential of random sequences to encode bioactive RNAs or peptides in bacteria (Keefe and Szostak 2001; Hayashi et al. 2003; Neme et al. 2017; Knopp and Andersson 2018) and higher organism (Carvunis et al. 2012; Chacón et al. 2014; Vakirlis et al. 2018; Heames et al. 2020; Vakirlis, Acar, et al. 2020). Regardless of the mechanism underlying their natural origin, the TRGs represent an interesting case in the evolution of genes—either as relics or a recent, lineage-specific innovation.

Understanding TRG function is difficult. The lack of homologs leaves researchers with two options: experimental validation or various ab initio predictions using available computational tools. Experimental procedures are laborious and low throughput, therefore, most of the functional annotation of TRGs is based on protein motifs and domain searches, and the characterization of general properties such as complexity or aggregation potential. Numerous studies have reported on the identification and characterization of functional TRGs from a variety of organisms [e.g., *Caenorhabditis elegans* (Zhou et al. 2015), the honey bee (Johnson and Tsutsui 2011), fruit fly (Zile et al. 2020), ash tree (Sollars et al. 2017), and primates (Toll-Riera et al. 2009)]. Some initial studies have been performed on bacteria (Fischer and Eisenberg 1999; Jordan et al. 2001; Siew and Fischer 2003a; Litman and Dishaw 2014) including *Escherichia coli* (Daubin and Ochman 2004; Yu and Stoltzfus 2012), *Xanthomonas campestris* (Chin et al. 2007), and *Acidithiobacillus* (Esposti 2018) due to quickly accumulating genomic data and the enormous diversity of molecular, morphological and ecological properties of bacterial species. These studies resulted in identification of a conserved structural motif in a species-specific protein (Chin et al. 2007), general association of orphan genes with bacterial pathogenicity (Entwistle et al. 2019) and specific aspects of development (Shi et al. 2020; Futo et al. 2021).

In this study, we investigated the origin, properties and function of TRGs in the *Bacillus* genus. *Bacillus* encompasses exceptionally diverse biotypes including human pathogens (e.g., *B. anthracis*), economically important entomopathogens (*B. thuringiensis*) (Baranek et al. 2020), species and strains used in industrial biotechnology (e.g., *B. subtilis 168*) (Su et al. 2020), and ecologically diverse nonpathogenic species (e.g., *B. mycoides*). However, previous studies mainly focused on the species *Bacillus subtilis* (Ravikumar et al. 2018) and concerned new genes involved in sporulation (Shi et al. 2020) and biofilm formation (Futo et al. 2021). In our study, as a primary source, we used the available at the time annotation of genes encoding proteins from all *Bacillus* species and removed false predictions with the aim of defining a core set of TRGs. By further exploring the noncoding space of the available genomic sequences, we identified corresponding fragments located both inside and outside of the genus, which may suggest a de novo origin for a large fraction of identified TRGs. We also explored the properties of TRG sequences and compared them with unannotated ORFs and the remaining fraction of the annotated proteins. Our findings indicated that TRGs in *Bacillus* display intermediate properties when compared with random ORFs and sequences with defined homologies. In addition, our exploration of the TRGs’ protein clusters indicated that some of them can form gene families restricted to the Bacillus taxonomic clade.

## Results

### Database Size, Taxon Composition, and Missing Gene Annotations Affect the Number of Predicted TRGs

The identification of TRGs and sequence homology prediction share similar methodological principles. However, in the latter case, the goal is to identify sequences that meet similarity criteria that can be used as a proxy for inferring shared ancestry. In contrast, when searching for TRGs, finding a homolog disqualifies the gene of interest. The most common tool used for identifying homologs and TRGs is the protein BLAST (BLASTp) program (Altschul et al. 1990), supplied with an appropriate *e*-value threshold. The *e*-value parameter depends on the size of the database. Hence the number of protein sequences available at the time of a BLAST search will directly affect the assignment of the query gene as either homologous or taxonomically restricted.

**Fig. 1.**
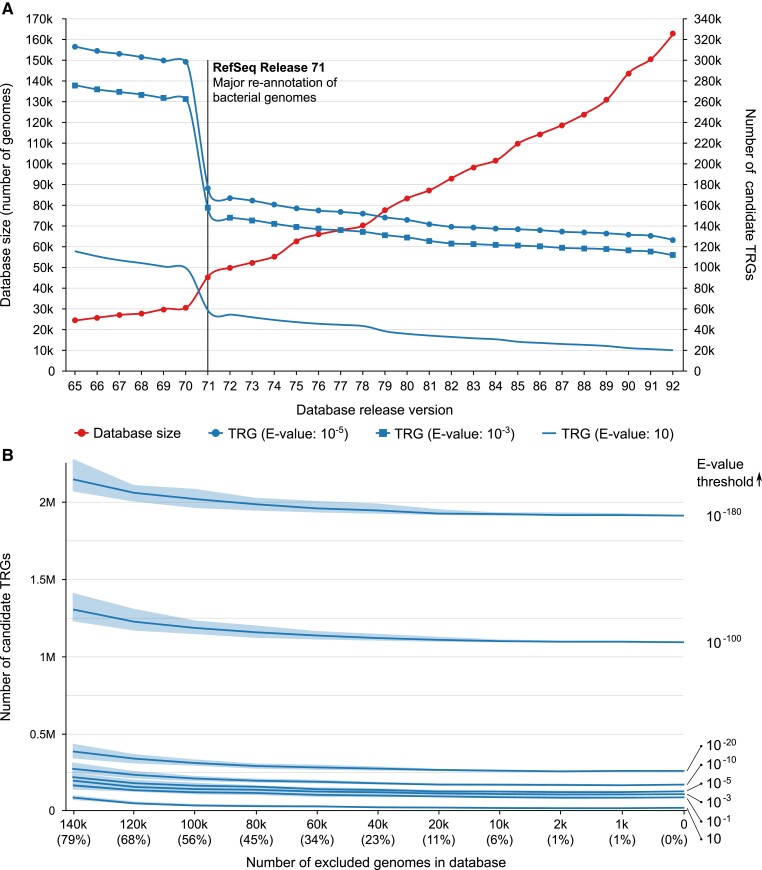
Putative genus-specific genes in Bacillus predicted using BLAST with (*A*) commonly used *e*-value cutoffs (i.e., 10^−5^ and 10^−3^) across 27 historical GenBank and RefSeq databases releases; (*B*) a broad range of *e*-value cutoffs across differently sized, randomly selected subsets of GenBank and RefSeq databases. The line marks the median number of putative TRGs. The bands show the minimum and the maximum number of putative TRGs.

We re-examined the influence of database size for relaxed (*e*-value = 10) as well as more stringent and commonly used *e*-values thresholds (10^−3^ and 10^−5^) (Siew and Fischer 2003c; Altenhoff et al. 2016; Ravikumar et al. 2018). The *Bacillus* complete proteome (represented by 396 *Bacillus* species with 2,826 assembled genomes containing 3,345,794 unique protein sequences; for details see Methods) was compared with bacterial sequences deposited in NCBI RefSeq and GenBank versions spanning four years. The number of putative TRGs identified using this approach is shown in figure 1. As expected, the increasing number of available bacterial genomes was negatively correlated with the number of predicted TRGs. The slope of the decreasing number of predicted TRGs was generally constant along the time scale with one exception; there was a large decrease in 2015 due to a major update of the RefSeq database (version 70) (fig. 1*A*). This update covered multiple changes to the bacterial genomes including completion of the re-annotation project and a transition to the new, nonredundant RefSeq protein data model (“RefSeq Announcements for 2015”). The improved annotation of genomic sequences supplied homologous sequences for more than half of the genes previously classified as TRGs. These homologs primarily came from unclassified species, followed by species from *Pseudomonas*, *Streptomyces*, *Clostridium*, *Lactobacillus*, and *Acinetobacter*. The analysis of historical GenBank and RefSeq database releases confirmed the influence of the database size and the major effect of the BLAST *e*-value threshold on the number of identified TRG sequences. Next, we examined the impact of taxon sampling on TRG identification efficiency. We included a randomized set of genomes along with a broader range of BLAST *e*-values (i.e., 10, 10^−1^, 10^−3^, 10^−5^, 10^−10^, 10^−20^, 10^−100^ and 10^−180^). After running 100 repetitions for each data set (for details, see Methods), we observed a similar effect to the previous analysis—a continuous decreasing slope of the number of predicted TRGs along with the increasing database size (fig. 1*B*). The observed small effect of the reference database subsampling clearly shows the superior effect of the *e*-value parameter over the database size. In addition, when compared with the historical data analysis (fig. 1*A*), it also points to the substantial effect of the reference database composition.

**Fig. 2.**
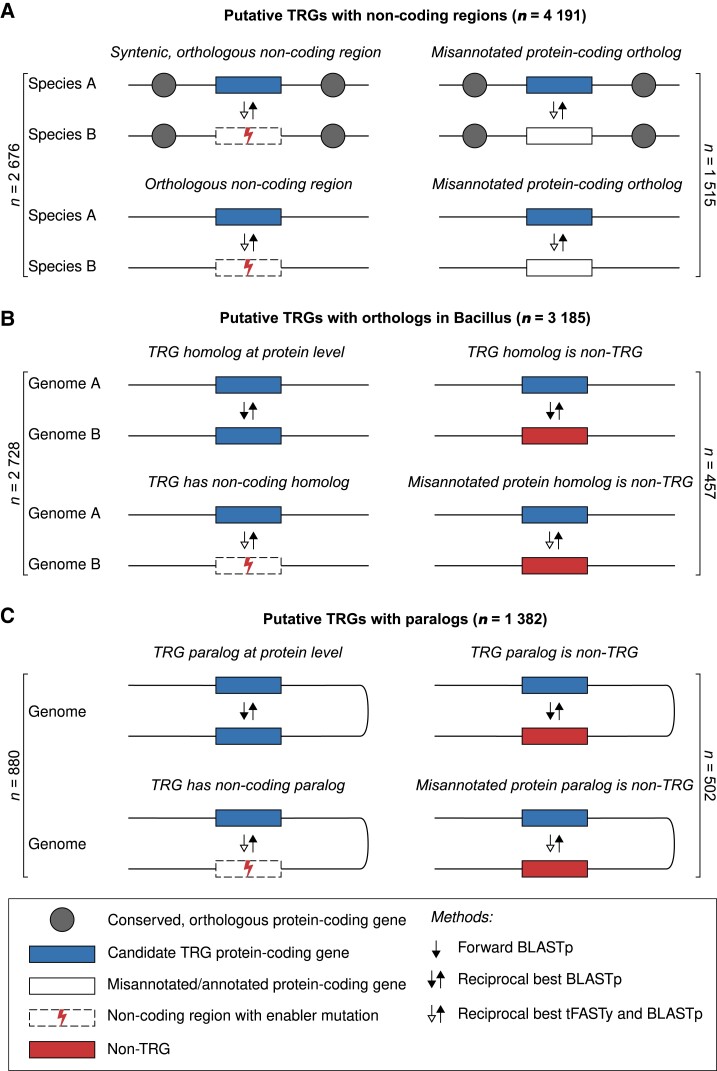
Graphical presentation of the classification schema applied during TRG identification. Proteins were classified as a TRG based on several different types of pairwise sequence comparisons. Based on the type and characteristics of the matching sequence, a protein could be classified as a TRG (left column) or a non-TRG (right column). The first classification step depends on the presence (non-TRG) or absence (TRG) of a detectable ortholog in a species outside of the Bacillus genus; (*A*) Classification based on the exploration of the noncoding syntenic (gray circles) and non-syntenic genomic regions; identification of nonannotated genes; (*B*) Exploration of homologs (coding and noncoding sequences) in genomic sequences belonging to the same species (strains); filtering of the putative TRG list is based on the restricted status of the homologous sequence; (*C*) Identification of paralogs (coding and noncoding sequences in the same genome) and assignment of restricted-gene status.

Some of the previous studies aimed at identification of the TRGs from various organisms included a step of *e*-value parameter optimization (e.g., Domazet-Loso (2003); Vakirlis, Carvunis, et al. (2020))) to achieve the desired rate of false-positive and false-negative predictions. Since the *e*-value seems not to be a very efficient parameter to retrieve TRG sequences, in our study, we have applied a different approach to minimize its use throughout the study. Therefore, instead of optimizing the single *e*-value threshold, we designed a multistep procedure to restrict the use of the *e*-value to only the first initial step. All subsequent parts of the identification procedure, which do not depend directly on this parameter, allow a fine reduction of the number of false-positive TRGs (supplementary fig. S1, Supplementary Material online). Because of the volume of the data (not comparable with previous studies) and complexity of our approach, we decided to use the most stringent *e*-value threshold of 10, thereby ensuring that while some of the true TRGs could be missed (reducing sensitivity), the main study goal would not suffer from extensive false-positive TRG predictions. Briefly, the procedure used a combination of sequence similarity searches at the DNA and protein levels (supplementary fig. S1, Supplementary Material online), where the *e*-value parameter is used only once throughout the whole procedure. Searches at the DNA level reduced the effect of missing protein annotations and allowed for the identification of candidate noncoding genomic fragments homologous to TRGs. The procedure further validated the candidate noncoding regions using the reciprocal best hit (RBH) approach (Altenhoff et al. 2016). Our procedure compared the protein sequence of a TRG with the corresponding noncoding DNA sequence using tFASTy (Pearson et al. 1997). The tFASTy tool allows to compare a protein sequence with peptides encoded by DNA that may be translated from different reading frames or disrupted by stop codons. In this way we could detect changes in homologous DNA that can impede its coding potential (e.g., stop codons and frameshifts). The procedure also inspected the syntenic context of identified TRG-related, noncoding regions by examining flanking genes. Finally, identified TRGs were grouped into gene families based on their orthologous and paralogous relationships (for details, see Methods). Overall, our multiplex procedure of TRG prediction led to the identification of TRGs by considering various evolutionary pathways (fig. 2).

To identify genus-specific TRGs, we created a nonredundant set of protein sequences from all (2,826) available *Bacillus* species (see Methods). These sequences were used in a BLASTp search against a complete collection of bacterial protein sets obtained from GenBank and RefSeq databases. We identified 5,268 putative TRG sequences specific to the *Bacillus* genus, which corresponded to 0.16% of all annotated *Bacillus* genes (supplementary Table S1, Supplementary Material online).

In subsequent steps, we explored the noncoding space of the bacterial GenBank and RefSeq genomes using the 5,268 putative TRGs as queries and the reciprocal tFASTy/BLASTp approach (fig. 2*A*). As a result, we have observed two types of matching sequences to genomes of species other than *Bacillus*: (i) fragments covering ≥90% of the TRG sequence and encoding a single, continuous ORF (coding) (fig. 2*A* right panel); (ii) regions containing frameshifts, premature stop codons, and sequences generating short reciprocal similarity hits (noncoding) (fig. 2*A* left panel). Nearly 29% of the putative TRGs (1,515 out of 5,268) matched (with query coverage of ≥90%) genomic regions encoding continuous ORFs uninterrupted by frameshifts and/or premature stop codons. The high level of similarity of these hits suggests that they were most probably classified as taxonomically restricted due to the poor quality of protein gene annotation and the non-*Bacillus* reference species containing the corresponding, homologous protein(s). Based on the status of the corresponding genomic region (coding and noncoding), we further filtered the list of putative TRG sequences (supplementary fig. S1 and Table S1, Supplementary Material online) assuming that, in the case of true TRGs, the corresponding genomic region from organisms outside of the *Bacillus* genus should contain obvious marks of the noncoding character (fig. 2*A*).

A parallel step in this TRG identification procedure included the search for syntenic DNA regions in reference bacterial genomes that corresponded to genomic fragments containing the TRGs (fig. 2*B*). At this stage, we used annotated, protein-encoding genes that flanked the putative TRGs as anchoring points to define corresponding genomic fragments in other species (see Methods). In total, we found almost one-third of genus-level TRGs (1,712 out of 5,268; supplementary Table S1, Supplementary Material online) with at least one corresponding syntenic DNA region in genomes belonging to genera other than *Bacillus*. By combining the results from both these analyses, we assessed the fraction of TRG sequences with corresponding syntenic regions that contained noncoding sequences showing the highest (reciprocal) similarity. The results showed that the extent of TRGs’ similarity to noncoding regions bypassed the boundaries of synteny, and that 18% (964 out of 5,268) of the putative TRG sequences displayed significant similarity to other, non-syntenic genomic regions from distant species. The species-specific genes represented a fraction of the genus-level TRGs and followed the general distribution trends observed for genus-level TRGs (supplementary Table S1, Supplementary Material online).

### TRGs Can Form Clusters of Homologous, Taxonomically Restricted Genes

To further explore the restricted character of the identified TRGs, we expanded the analysis to identify homologs of TRG sequences (orthologs and paralogs) on the (i) genus (between species), (ii) species (between genomes), and (iii) genome (within the same genome sequence) levels. For this step, we selected 3,753 TRG sequences (supplementary Table S1, Supplementary Material online) that showed no similarity (1,077) or a similarity to only noncoding regions (2,676) in the reciprocal search query against the entire bacterial GenBank and RefSeq datasets. A summary of the results is provided in supplementary Table S2, Supplementary Material online.

The reciprocal BLASTp and tFASTy/BLASTp searches for genus-level TRG homologs across *Bacillus* species resulted in the identification of orthologs (annotated proteins or nonannotated genomic regions; fig. 2*C*) for nearly 85% of the genes (3,185 out of 3,753) (supplementary Table S2, Supplementary Material online). Surprisingly, close to 12% (457) of the identified TRGs had orthologs that could not be classified as taxonomically restricted (fig. 2*C* right panel; supplementary Table S2, Supplementary Material online). Therefore, the genes with such a transitive homology to a non-TRG sequence were removed from the TRG dataset as false-positive predictions. Conversely, we found homologous sequences for only eight species-level TRGs (out of 75), which implied a very restricted distribution of these sequences within genomes belonging to the same species. All these TRG-matching sequences were classified in previous steps as taxonomically restricted (supplementary Table S2, Supplementary Material online). Finally, our search for TRG paralogous sequences (within the coding and noncoding sequences) led to the identification of 1,382 sequences that formed gene families (fig. 2*D* left panel). However, almost 36% of them (502) were not classified in previous steps as taxonomically restricted (fig. 2*D* right panel). Similarly, out of three species-specific TRGs with corresponding paralogs, only one was not classified as a TRG. Following the filtering rules applied in the previous step, we removed these putative TRGs from further analysis (supplementary Table S2, Supplementary Material online). One possible explanation of the large number of TRG's homologs (orthologs and paralogs) that were not classified as taxonomically restricted could be the presence of gene fusions that may link sequences similar to TRG homologs with annotated genes. However, our search for such fused genes showed that they might account for no more than 1% of the cases (data not shown).

The verification procedure for the restricted status of TRG homologous sequences further reduced the number of predicted TRGs to 2,794 genes (0.08% of the original 3,345,794 complete set of *Bacillus* annotated, nonredundant proteins). Among them, 71 could be identified in only one species. The highest number of species-specific TRGs was found in *B. anthracis* (13) followed by *B. cereus* (5), *B. coagulans* (4), and *B. thuringiensis* (4). In the remaining 38 species, the number of orphan genes oscillated between 1 and 3 (supplementary Table S3, Supplementary Material online). These TRG-containing species are spread across *Bacillus* phylogenetic tree (supplementary fig. S2, Supplementary Material online) and different bacterial groups, which represent various morphological (e.g., colony characteristics, cell diameter), biochemical (e.g., hydrolytic capabilities) and physiological properties (e.g., optimal pH, NaCl concentration and temperature growth conditions). We could not observe any obvious correlation between their biological properties and the distribution of TR genes. No species-specific genes were found in 354 *Bacillus* species. However, based on the very stringent identification parameters, these numbers most likely represent the lower limit of the TRG sequences in *Bacillus*.

**Fig. 3.**
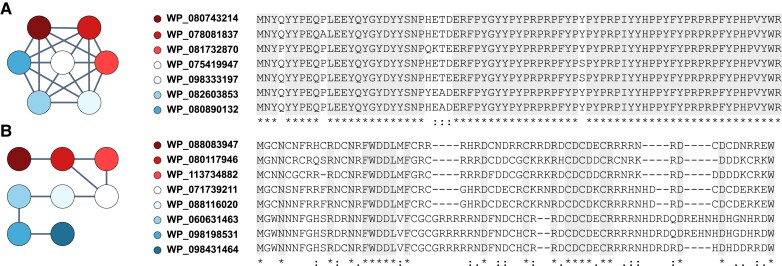
Examples of the orthology graphs (Kuzniar et al. 2008; Altenhoff et al. 2013) of TRG clusters as determined by the reciprocal best BLAST hit method: clusters of highly similar (*A*) and more divergent (*B*) sequences. All of the clusters show various combinations of these two exemplary topologies (all clusters’ topologies containing more than 2 sequences are shown in supplementary fig. S3, Supplementary Material online).

Using the information on homologous relationships (reciprocal similarity best hit results) between TRGs, we clustered them using the single-linkage approach into *Bacillus* TRG families. The sequences formed 2,464 distinct groups with most of them (98%) containing only one (2,311) or two (102) sequences. However, some of the TRGs were grouped into larger clusters, with the largest containing 34 sequences. The genes forming this cluster exhibited a high level of identity and were distributed amongst 34 *Bacillus* species (supplementary Table S4, Supplementary Material online). The remaining TRGs formed groups composed of 3 to 15 sequences. A closer inspection of the clusters’ node connection structure revealed that they were built with highly similar sequences (fig. 3*A*) and more divergent proteins often linked to single nodes (fig. 3*B*). The cluster connection topology well represents sequence conservation, where fully connected clusters are composed of almost identical sequences and sparsely distributed groups are composed of more divergent proteins. However, most of the identified clusters of homologous TRG sequences showed mixed properties (supplementary fig. S3, Supplementary Material online), where proteins are connected by a variable number of edges representing the best reciprocal similarity hits. The species distribution of the *Bacillus* TRG homologs indicated that nine of them may form paralogous pairs (supplementary Table S5, Supplementary Material online). We identified the highest number (4 pairs) of such paralogs in *B. cereus*. Interestingly, in one of the *B. cereus* genomes (GCF_900095125) we found two paralogous pairs. The other homologs were located in the genomes of *B. thuringiensis*, *B. toyonensis, B. sp. FJAT-45086* and *B. sp. 491mf*. The sequence similarity between the paralogous pairs varied between 35% and 100%.

The identified clusters of TRG homologs show that at least some of these genes expand by forming gene families in a similar way to widely taxonomically distributed, older genes. Similarly, different patterns of sequence conservation (fig. 3) may indicate distinct modes of functional adaptation. However, in the case of TRGs in a much more confined space of closely related organisms classified to the same genus or species.

### For a Significant Fraction of Bacillus TRGs Only Small Sequence Changes Separate Noncoding Regions From Coding Genes

**Fig. 4.**
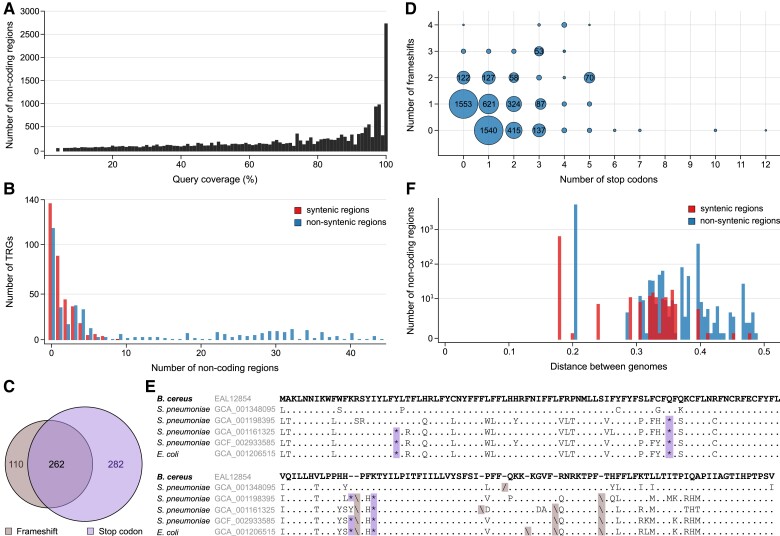
Noncoding homologous sequences of Bacillus TRGs in non-Bacillus genomic sequences. (*A*) Distribution of TRG sequence coverage with noncoding genomic regions in genomes from species outside of the Bacillus genus. (*B*) Distribution of high coverage (≥90%), noncoding fragments mapping to one TRG sequence within or outside of syntenic regions. (*C*). Venn diagram showing the distribution of two types of sequence variability (frameshift and premature stop codon) disrupting the coding potential of TRG-mapped, noncoding regions. (*D*). Groups of noncoding fragments containing various numbers of stop codons and/or frameshifts. (*E*) TRGs in *B*. *cereus* that are orthologous to noncoding genomic regions in *S. pneumoniae* and *E. coli*. (*F*) Distribution of the genomic distances (as reported by Mash (Ondov et al. 2016)) between genomes containing TRGs and corresponding genomes with noncoding fragments. The count representing each distance is shown in logarithmic scale.

During the identification of putative TRGs, we found a high fraction of TRG sequences (71%; 1,975 out of 2,794) that had at least one corresponding, noncoding fragment located in other bacterial genome(s) that were external to the Bacillus genus (EBG). These regions exhibited a wide range of similarities (from 44% to 100%) and diverse sequence coverage (fig. 4*A*). Among them, 739 TRGs (37%) showed at least one high-coverage (≥90%) matching fragment located within (331) or outside (408) of the syntenic region(s). In total, these high-confidence, noncoding matches constituted more than 48% (6,904) of all TRG-related, EBG, noncoding fragments (14,268).

On average, one TRG sequence was mapped to nine noncoding, high-coverage fragments in the tested EBG genomes (2 and 15 for syntenic and non-syntenic noncoding regions, respectively; fig. 4*B*). The highest number of TRGs mapped to a single syntenic or non-syntenic noncoding region. The non-syntenic matching regions dominated the distribution along with the increasing number of corresponding noncoding matches. Such a pattern may be associated with the loss of synteny conservation in the genomes of species distant to *Bacillus* or with decreasing efficiency of syntenic region detection (Vakirlis, Carvunis, et al. 2020). For example, the highest number (363) of non-syntenic, noncoding fragments corresponding to one TRG sequence was found in the genomes of *Clostridioides* (359), *Clostridium* (3), and *Streptococcus* (1) genera. The number of noncoding fragments identified within syntenic regions was significantly lower (*P* = 7.54 × 10^−25^; Mann–Whitney U test), showing a maximum of 10 fragments mapping to one TRG sequence.

The identification of homologous, noncoding sequences provided an opportunity to explore sequence changes that alter coding capacity in homologous regions (nonsense mutations/stop codons and indels/frameshifts). We investigated stop codons and frameshift sequence changes for each pair of a TRG and corresponding noncoding fragment. Figure 4*C* shows that more than one-third of the TRGs (35%; 262 out of 739) matched noncoding fragments that carried both stop codon and frameshift sequence changes. Interestingly, the second most frequently observed (31%; 228 out of 739) sequence change responsible for disrupting the coding potential of the homologous genomic regions was the stop codon, followed by frameshifts, which accounted for 15% (110 out of 739) of the changes. The pattern of dominant sequence changes leading to premature stop codons is visible in figure 4*D*, which shows a correlation between the number of frameshifts and the number of the stop codon sequence changes. In one extreme case, we observed a region where the coding capacity was interrupted by 12 stop codons (fig. 4*D*). Similarly, the most extreme example of a frameshift sequence change was a fragment with four modifications (fig. 4*E*). An extreme example of noncoding fragments carrying both types of sequence alterations included regions with five stop codons and four frameshifts. However, the dominant fraction of the noncoding fragments carried only one of the mutations (1,540 frameshifts and 1,553 stop codons). This group of fragments may represent a reservoir of noncoding sequences that can be transformed into coding genes by a single nucleotide change. Inversely, the decreasing count of fragments with a higher number of sequence changes disrupting coding capacity may indicate the effect of rapid evolutionary turnover of coding potential (Nielly-Thibault and Landry 2019).

We also investigated the taxonomic distribution of bacteria containing TRG-corresponding, noncoding genomic sequences by comparing the distances of the source (TRG-containing genome) to the target genomes containing the related noncoding fragment(s). Figure 4*F* shows the distribution of the whole-genome distance for high-coverage fragments mapped by each of the 739 TRGs. In total, the target genomes represented 117 genera (38 for syntenic and 79 for non-syntenic matches). In figure 4*F*, two dominant peaks coming from genomes belonging to the *Streptococcus* genus can be seen, which are followed by similar overlapping signals with an increasing tendency towards a higher number of non-syntenic fragments along with a growing distance. The full list of all taxonomic groups containing high-coverage, noncoding sequences is provided in supplementary Table S6, Supplementary Material online.

The presence of noncoding regions corresponding to TRGs in distantly related bacteria may point to the genetic source for these genes. However, it may be that these regions are the result of a diverging coding sequence, that is a protein-coding sequence that escaped purifying selection and is randomly changing creating a gene-like (pseudogene) element. This scenario is plausible for a one-to-one relation between a TRG and a noncoding region. However, in the case of one TRG and many noncoding syntenic DNA fragments, the explanation of molecular and evolutionary mechanisms leading to the preservation of the coding capacity of a gene in an isolated lineage may be more complex.

In the case of species-specific TRGs (71 sequences) we could not identify any corresponding noncoding fragments between *Bacillus* species, within species (different genomes), or inside the genome. The species-level, TRG-corresponding, noncoding regions could, however, be found outside of the *Bacillus* genus: three for non-syntenic and two for syntenic regions. In contrast, the remaining genus-level TRGs were well represented in available *Bacillus* sequences with noncoding fragments that were identifiable for almost 96% of the TRGs (2,607 out of 2,794).

### TRG Sequences Show Distinct Properties From ORFs and Annotated Proteins

The vast majority (>90%) of the 2,794 *Bacillus*-specific TRGs were annotated as a “putative protein”, “uncharacterized protein” or “protein with a domain of unknown function” (supplementary Table S7, Supplementary Material online). To better characterize these proteins, we analyzed their sequence properties (length, complexity, and disorder) as well as their ability to aggregate or encode known functional protein domain(s).

**Fig. 5.**
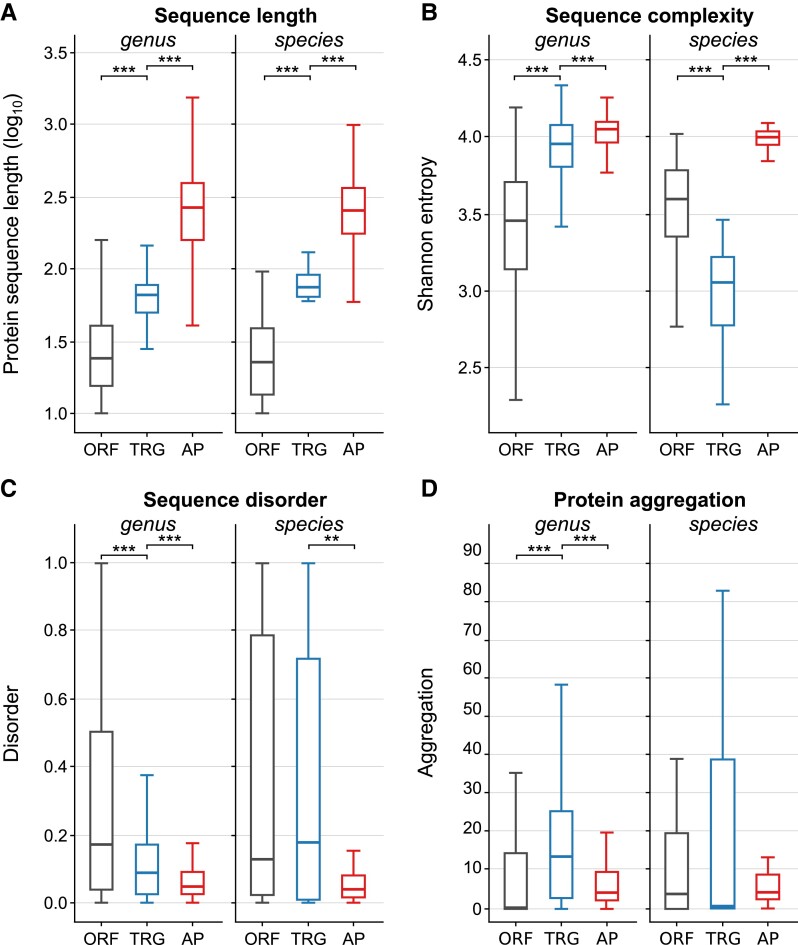
Properties of protein sequences of Bacillus taxonomically restricted genes (TRGs) at genus (n = 2,794) and species (n = 71) levels. TRGs (blue boxes) are compared with randomly selected, equal to TRG count, subsets of nonannotated open reading frame (ORF) sequences (gray boxes) and annotated non-TRG proteins. (*A*) Protein sequence length (*B*) sequence entropy (*C*) Protein disorder (*D*) aggregation potential normalized to the protein length (see Methods). Boxes indicate interquartile ranges and medians (horizontal line within each box). Whiskers indicate minimum and maximum values. Asterisk mark statistical significance of Wilcoxon signed-rank test at *P* < 10^−10^ (***), *P* < 10^−3^ (**), and *P* < 10^−2^ (*).

The length of the TRG protein sequences identified at the genus and species levels were compared with the lengths of well-annotated genes and ORF sequences that did not overlap with annotated features (see Methods). As shown in figure 5*A*, the protein sequences of TRGs detected at both levels tended to be longer than nonannotated ORFs and shorter than annotated (non-TRGs) protein sequences (*P* < 2.2e-16; Wilcoxon test). Specifically, the median sequence length of genus and species-specific TRG proteins (67 and 74 amino acids, respectively) was two to three times greater than the median length of ORF sequences (23 and 44 amino acids) and three to four times lower than the length of annotated, non-TRG sequences (251 and 274 amino acids).

Next, we analyzed the sequence complexity of TRGs in reference to ORFs and annotated genes using Shannon entropy (see Methods). We observed clear differences between the distinct types of sequences in the distribution of the entropy values (*P* < 2.2e-16; Wilcoxon signed-rank test) (fig. 5*B*). Interestingly, genus-level TRG median entropy values (3.96) were positioned closer to annotated proteins (4.05) than to nonannotated ORFs (3.46), indicating a notable level of TRG sequence complexity. In contrast, the distributions of Shannon entropy for TRG proteins at the species level had the lowest values (fig. 5*B*). The median for TRG proteins (2.55) was much smaller (*P* < 6.16e-12) than the corresponding values for ORFs (3.41) and annotated proteins (4.04).

Recent (young) genes encode for proteins with a high content of intrinsically disordered regions (Wilson et al. 2017) that adopt a highly flexible conformational ensemble rather than a single, well-defined structure. Therefore, we examined the disordered character of *Bacillus* TRGs using the IUPred tool (Dosztányi 2018) and calculated an overall disorder score (between 0 and 1). In the case of genus-level TRGs, we observed (fig. 5*C*) a clear distinction (*P* < 2.2e-16) in the distribution of disorder scores when compared with ORF and annotated protein sequences. The TRGs were positioned (median 0.09) between nonannotated (median 0.17) and annotated sequences (median 0.05), thus showing a tendency towards more ordered, well-defined structures. The analysis of species-level TRGs displayed again a disparity as already seen in the sequence complexity estimation (fig. 5*B*). The ORF and TRG scores clearly differed from those obtained from annotated proteins (*P* = 3.0e-4) (fig. 5*C*). However, the score distributions of the ORFs and TRGs were not statistically different, and their median values (0.13 and 0.18, respectively) were three to four higher than the median of annotated proteins, thus indicating a tendency of these proteins towards a higher content of flexible structures. In addition, both types of proteins exhibited a much wider spectrum of score values when compared with annotated proteins (fig. 5*C*). It has been previously shown (Ángyán et al. 2012) that sequence GC content may directly affect the intrinsically disordered character of proteins. To investigate this effect in the case of *Bacillus* TRGs, we plotted the genome GC content for the disorder score of species-level TRGs. As shown in supplementary figure S4, Supplementary Material online no such unequivocal correlation in this case could be detected.

Protein aggregation is regarded as a mechanism that hampers proper protein folding and often induces cytotoxicity. It is essentially a self-association process by which identical protein molecules form higher-order conglomerates of low solubility that eventually precipitate. Thus, functional proteins have often evolved strategies to minimize aggregation (Monsellier and Chiti 2007). The mechanism of protein-aggregate toxicity remains unclear, but accumulating evidence suggests that it is related to the interaction between the aggregates and the cell membrane (Rousseau et al. 2006). Using the Tango prediction server (Fernandez-Escamilla et al. 2004), we assessed the aggregation potential of all three tested datasets. Interestingly, the distribution of the average aggregation scores (fig. 5*D*) placed the genus TRG encoded proteins as the most likely to aggregate in comparison with ORFs and annotated proteins. Although this tendency is reversed in the case of species TRGs (lowest average aggregation score), the distribution of calculated scores covers a wider range of values. These results may indicate an unusual (not present in ORF and annotated sequence) tendency of TRG to form aggregates.

In three out of four analyzed properties of TRG proteins, we observed unexpected differences between genus and species sequences. Although the observed variability may be due to the specific properties of these proteins, it has to be noted that it may also be the result of small sample size (71 sequences) in the case of species TRGs.

Although TR genes are generally of small size, contain low complexity sequence regions, and show a high tendency to form aggregates, we investigated the presence of protein functional motifs. As expected, we did not find any clearly defined, specific for a particular molecular process protein domains suggesting that TRGs mainly represent novel genes that are in the process of acquiring molecular function. About half (1,456; 52%) of the TRGs showed presence of the nonspecific, general type of motifs, including non-cytoplasmic/cytoplasmic domains (1,161; 80%) and transmembrane domains (1,139; 78%), followed by signal peptides (260; 18%) and structural (coil and disordered) motifs (76 and 184, respectively). We did not observe any significant differences in the domain distribution between genus and species-level TRGs, nor between TRGs with or without syntenic blocks.

The above analyses show that when size, sequence complexity and intrinsic disorder (on the genus level) are considered, the identified TRGs represent intermediate states between nonannotated ORFs and annotated, functional proteins. However, the potential to form protein aggregates seems to be a specific feature of proteins encoded by taxonomically restricted genes. The properties of species-level TRGs do not, in most cases, follow the characteristics of genus TRGs. However, because of the small sample size of the species-specific TRGs, it is difficult to draw final conclusions.

## Discussion

The identification of proteins restricted to a taxonomic group depends on the same principle as homolog identification; however, the goal is the opposite—that is, to find sequences without detectable evolutionary relatives outside the group. In both cases, one of the most important steps is the choice of appropriate search parameters and a representative database of reference sequences. This study confirmed the pivotal role of BLAST parameters (*e*-value) and additionally showed the influence of database content used for sequence similarity searches. By examining historical data, we were able to demonstrate that RefSeq release 71 dramatically decreased the number of predicted TRGs following a major update of bacterial genome annotations. This effect was further amplified by the application of various *e*-value thresholds. In contrast, recent database volume changes did not influence TRG prediction outcomes to the same extent. These results point to the crucial influence of the taxonomic representation within the database rather than its volume. Additionally, we showed that the number of predicted TRGs has stabilized since RefSeq release 71, with TRGs gradually declining with the growing number of available genomes.

A more substantial and stable effect on the quality of TRG prediction stemmed from the selection of the sequence similarity threshold. In our study, we tested the BLAST *e*-value, with a higher *e*-value signifying a more restrictive search for unique TRGs. A poor choice for this parameter could produce a number of false predictions that is proteins that have homologs but are classified as taxonomically restricted. We observed a distinct and constant difference between the most commonly used *e*-values in previous studies: 10^−5^ and 10^−3^. Even a small change in this parameter produced an enormous difference in the number of predicted TRGs.

Based on these results, we conclude that there is no universal standard value that can be selected for TRG identification. In our opinion, this parameter should be selected depending on the type and the amount of available data as well as the goal of the study. In our case, we were interested above all in identifying true TRG sequences, even at the cost of losing sensitivity. Therefore, we selected the highly restrictive *e-*value of 10, and the number of TRGs identified likely represents the lower bound of the real number in the *Bacillus* genus. Although the selection of such a high *e*-value parameter may implicate exclusion of true restricted genes just by chance, we were still able to identify a notable fraction of *Bacillus* genes as TRGs. Moreover, in subsequent steps the predicted candidates were further reduced (almost by 50%), indicating that a significant portion of the initially annotated at such high *e*-value genes were still false positives. In our opinion, expanding a search for TRGs by lowering the *e*-value parameter should be accompanied by additional evaluation procedures such as transcription and/or translation status estimation, which would allow for a clear distinction between true and false predictions.

We expanded the TRG identification procedure by adding steps that allowed us to further filter false predictions concerning the origin of *Bacillus* TRGs. In particular, searching the noncoding space of the bacterial genomes with the TRG protein query resulted in the identification of many sequences that were missed by annotation in some of the reference genomes. The problem of missing genes in the annotation of the prokaryotic genomes has been previously addressed (Warren et al. 2010; Wood et al. 2012), showing that it largely affects identification of short sequences. The effect of a method used during genome annotation on lineage-specific genes identification has been also recently investigated for selected groups of vertebrates (Weisman et al. 2022). In addition, this step allowed us to identify noncoding genomic regions that resided in bacteria outside of the *Bacillus* genus. Interestingly, most of these regions possessed coding capacities and only small changes in the sequence separated them from encoding homologous to TRG proteins.

Two major mechanisms have been proposed for the origin of TR genes: i) small changes in the DNA transform a noncoding region of the genome into a functional, protein-coding ORF, or ii) the gene is a result of a copying and diversification mechanism within the same genome. Therefore, we extended our analysis to explore the diversity of sequences within *Bacillus*. As a result, we were able to define TRG families identified by the best reciprocal similarity group of sequences specific for the genus. We also found putative paralogs of TRGs. Our results suggest that the dominant mechanism of TRG origin in *Bacillus* occurs via de novo gene evolution from noncoding sequences. In this context, however, a TRG sequence is simply a step in the de novo evolution of a new gene. As a TRG enters the selectional environment it may transform into a new gene, spread across the genome (strains and species), or fuse with other sequences to create new functional units (Bornberg-Bauer et al. 2021). In support of this mechanism, it has been previously shown that random sequences are an abundant source of bioactive RNAs or peptides (Neme et al. 2017; Bhave and Tautz 2021; Castro and Tautz 2021). In addition, some of the TRG sequence properties, when compared with annotated proteins and ORFs, further support the conclusion of a noncoding sequence origin for the majority of TRGs in *Bacillus*.

In a similar way to several previous studies (e.g., (Carvunis et al. 2012; Basile et al. 2017; Vakirlis et al. 2018; Heames et al. 2020; Castro and Tautz 2021)), we tested the most obvious parameters such as length, complexity, disorder, and aggregation potential. As expected and complementary to other studies, on the genus level annotation three out of four tested attributes (sequence length, complexity, and disorder) located TRGs between annotated proteins and products of nonannotated ORFs. Most probably due to small sample size, the species-level TRGs do not follow this pattern in case of sequence complexity and disorder. In contrast, the analysis of protein aggregation properties of TRGs showed that they may represent a distinct group of proteins. This finding may appear contradictory to the hypothesis (Wilson et al. 2017) that proteins are optimized against aggregation during evolution. One can speculate that certain steps in the evolution of the novel protein core and transmembrane domains can render the proteins more prone to aggregation, and the selection to lessen this effect can operate after the suitable structure and stability are reached (Ángyán et al. 2012).

It has to be noted that our analyses concentrated on exploring the most common trends for these properties. When examining the data, we observed a large amount of overlap between the ORF sequences and TRGs. The same was also true for annotated proteins and ORF-derived proteins. Nevertheless, we believe that identification of such subtle differences between groups of these distinct genes was possible by application of very stringent annotation criteria implemented into our pipeline.

One important question remains: are TR genes functional, or do they only mimic functional genes? Our computational analyses indicated some subtle patterns of protein domains (namely non/cytoplasmic and transmembrane domains, and signal peptides) that were dominant within TRGs. These features may indicate that the TRGs are involved in specific molecular processes that could provide immediate results in adaptations to the environment (Vakirlis, Acar, et al. 2020). Hence, one would expect that the emergence of new TRGs should be evident under stressful conditions. Since TRGs are reported in many studies to be expressed in specific conditions (e.g., An et al. (2000); Ellrott et al. (2010); Shi et al. (2020)) and very often at low levels (e.g., Selinger et al. (2000); Ekman and Elofsson (2010); Yu and Stoltzfus (2012)), it might be very difficult to explore their activity using non-dedicated data sets (e.g., RNA-seq or mass spectrometry). Hence, the answers to these questions most probably will require carefully designed experimental work.

Concluding, we have designed and carried out a comprehensive TRG identification pipeline using available bacterial genomic sequences from the *Bacillus* genus. Our extensive comparative analyses strongly point to a de novo mode of majority of TRG evolution. In addition, we have explored the within-genus evolutionary properties of TRGs, which shows that, similar to annotated proteins, they may form gene families. Finally, the exploration of TRG sequence properties in most cases places them between nonannotated ORFs and annotated sequences with some very interesting exceptions.

## Methods

### Sequence and Taxonomy Data Sources

Genome assemblies, protein sequences, and annotation files for bacterial accessions, including 396 species from the *Bacillus* genus, were downloaded in December 2018 from the NCBI GenBank/RefSeq (O’Leary et al. 2016) databases, Release 90. Only genomes annotated from least at the scaffold level were analyzed. A total of 2,826 genomes belonging to 396 *Bacillus* species were processed, including 223 genomes assembled at the scaffold level, 494 complete genomes, and 109 chromosome-level assemblies. To reduce the volume of data for computational analyses, the complete, nonredundant set of the *Bacillus* proteins was assembled from the annotation data by clustering identical sequences. The reference protein sequences representing the Bacteria domain were downloaded from UniProt (Release 2018_09) (UniProt Consortium 2019) and the NCBI Genbank/RefSeq databases. Taxonomic information was obtained from the NCBI taxonomy database in December 2018 using the ETE3 Python programming toolkit (v. 3.0.0b34) (Huerta-Cepas et al. 2016).

### Sequence Similarity and Homology Searches

Sequence similarity searches at the protein level were performed using NCBI BLAST + (v. 2.7.1) (Camacho et al. 2009). Putative TRG proteins were compared with DNA sequences using the tFASTy program (v. 36.3.8 g), which is part of the FASTA3 toolkit (Pearson et al. 1997). This tool compares a protein with a DNA sequence translated in six frames. It allows for the identification of the best corresponding peptide regardless of the changes introduced by nonsense mutations and/or insertions/deletions. Hence, the use of tFASTy allows identification of protein-coding sequences that were missed (e.g., due to a frameshift) in the initial search by BLAST + tool. Homologous pairs of sequences (protein vs. protein and protein vs. DNA) were estimated using the RBH approach (Hirsh and Fraser 2001; Altenhoff et al. 2016). Putative TRG proteins were used in the first forward search to scan bacterial proteins (BLASTp) or genomic sequences (tFASTy). The sequences producing the best similarity hits (identified by score value) for each TRG protein were subsequently used as queries in reverse searches with all proteins from the queried bacterial proteome.

The homologous relations between TRG proteins defined by the RBH approach were used for clustering the sequences into gene families using the single-linkage approach implemented in Cytoscape with default parameters (Shannon et al. 2003).

### Estimation of the Effects of Database Size and BLAST *e*-value on TRG Detection

To explore the effect of database size on the identification of TRGs, the sequences representing all *Bacillus* proteins were searched against the Genbank and Refseq datasets with the BLASTp algorithm using three different *e*-value thresholds: 10, 10^−3^, 10^−5^. Information about the release date of a genome was extracted on April 17, 2020 from the list provided by NCBI at https://www.ncbi.nlm.nih.gov/genome/browse/#!/prokaryotes/.

The effect of *e*-values on the number of TRGs was estimated using a randomized set of genomes. Each data set was created by random exclusion of 1k, 2k, 10k, 20k, 40k, 60k, 80k, 100k, 120k, and 140k genomes from the complete genome list. The following BLAST *e*-values thresholds were tested: 10, 10^−1^, 10^−3^, 10^−5^, 10^−10^, 10^−20^, 10^−100^, and 10^−180^. The calculations were repeated at least 100 times for each randomized data set and each *e*-value threshold.

### Identification of Taxonomically Restricted Genes

The nonredundant set of *Bacillus* protein sequences was used as a query in searches with the Uniprot Bacterial Complete Proteome database using BLASTp with an *e*-value threshold of 10. Sequences without any matches/similarities were further queried against the entire NCBI Genbank and Refseq proteomes with the same *e*-value parameter. Proteins with no detectable similarity to non-*Bacillus* bacterial proteins were considered to be encoded by TRGs at the genus level.

To identify syntenic regions between *Bacillus* harboring putative TRGs and other bacterial genomes, we used the RBH approach. The protein sequences flanking a putative TRG were searched using reciprocal BLASTp searches to define a corresponding syntenic genomic region. Noncoding syntenic regions were searched by the tFASTy algorithm to identify putative peptides coded by sequences altered by nonsense mutations and/or frameshifts. To verify the homologous correspondence of these peptides, a backward BLASTp search was performed with the entire protein set encoded by the TRG-containing *Bacillus* genome. The tFASTy/BLSTp reciprocal search procedure was applied with the entire genomic sequence of the target bacterial genome in cases where the syntenic region could not be found. This procedure allowed us to identify TRGs with (fig. 2): (i) no detectable similarity; (ii) corresponding, nonannotated proteins (which were further discarded from the study), and; (iii) corresponding noncoding regions located within syntenic or non-syntenic regions. The tFASTy results representing the last group were further used to characterize the mechanisms involved in breaking the coding capacity of the noncoding genomic fragments.

The same reciprocal best hit procedure was applied in searches for homologous sequences in the coding/noncoding sequences among *Bacillus* species. For species-level TRGs, we searched the genome sequences within strains. This step allowed for the identification of TRG orthologs among *Bacillus* species and noncoding regions that exhibited homology to the TRG sequences. The exploration of TRG homologs was further expanded with reciprocal searches for sequences within the TRG-containing genome. For the identification of protein sequences, a BLASTp search was used in both directions. For noncoding paralogous sequences, the annotated protein-coding region in the genome was masked using the maskfasta module from the bedtools package (Quinlan and Hall 2010), and a tFASTy/BLASTp reciprocal search was then performed. Using this approach, we could identify TRGs with: (i) no homologs; and (ii) paralogs and noncoding homologous regions within the same genome. The identification of homologs within genus (orthologs) and genome (paralogs) and the evaluation of their status (taxonomically restricted or not) allowed us to further curate the list of TRGs in *Bacillus*.

Whole-genome sequence distances between bacterial species were calculated using Mash (v. 2.3) (Ondov et al. 2016) with a default *k*-mer size of 21 nucleotides and a 100,000-sketch size.

### Analysis of TRG Sequence Properties

The sequence properties of the TRGs were compared with annotated proteins and nonannotated peptides encoded by ORFs. As each of the datasets varied substantially in size, the same number of sequences originating from the same proteome were randomly selected for each reference dataset. Shannon entropy was calculated for each protein sequence using the SciPy Python package (Virtanen et al. 2020). The level of intrinsically disordered regions in protein sequences was calculated using IUPred2A-short software (Mészáros et al. 2018)). The number of residues with a disorder score above the threshold of 0.5 was divided by sequence length to give a disorder score for each protein in the datasets. Protein average aggregation was calculated using the statistical mechanics algorithm TANGO (v. 2.3.1) (Fernandez-Escamilla et al. 2004) and presented as a frequency of potential aggregating segments defined as hexapeptides with an aggregation score above 5% over all amino acid residues. Protein domains and motifs were predicted using InterProScan 5 (Blum et al. 2021) with default parameters.

## Supplementary Material

evad023_Supplementary_Data

## Data Availability

All data generated in this study are available in the Supplementary Information. The code used in the study is available on the Github server at https://github.com/Deeptivarshney/Bacillus_trg
