# Supplementary material for: Taxonomically Restricted Genes in Bacillus may Form Clusters of Homologs and Can be Traced to a Large Reservoir of Noncoding Sequences
Source: Genome Biol Evol. 2023 Feb 15;15(3):evad023. doi: 10.1093/gbe/evad023 (PMC10003748; doi:10.1093/gbe/evad023)
Supplement: evad023_Supplementary_Data [file evad023_supplementary_data.zip › Supplementary_Figures.pdf]

Taxonomically Restricted Genes in Bacillus may form clusters of homologs and can be traced to a large reservoir of noncoding sequences

Wojciech M. Karlowski*, Deepti Varshney and Andrzej Zielezinski

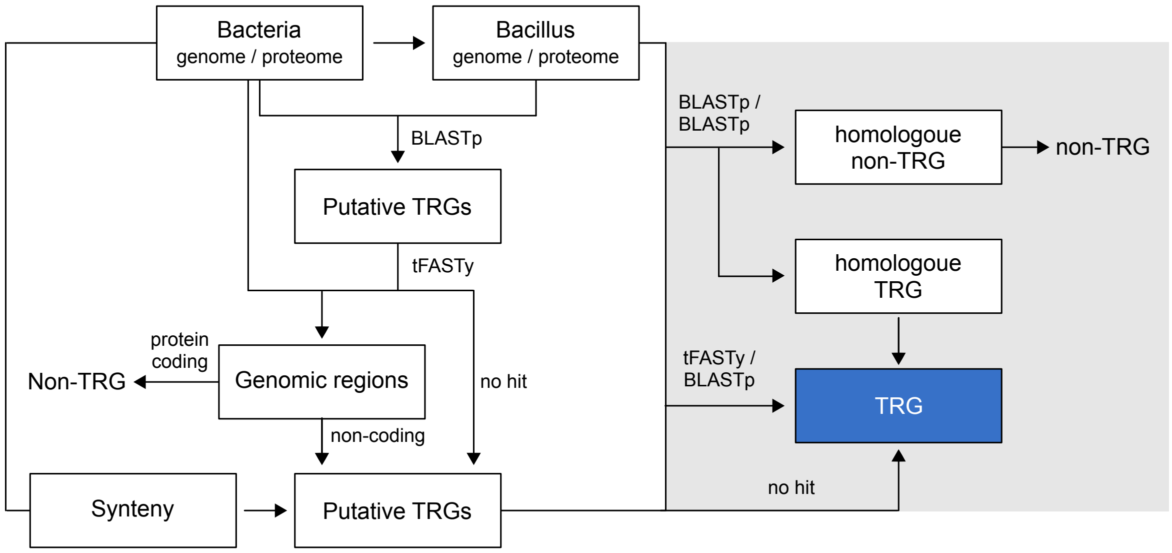

Figure S1. Schematic representation of the major steps involved in the identification of the TRG sequences. The left panel shows the typical BLAST-based approach supplied with the search of non-coding sequences with tFASTy algorithm. The right (grayed) panel presents steps implemented to identify within genus TRG homologs. Putative TRGs are identified in protein BLAST (BLASTp) searches between proteomes of *Bacillus* species and proteomes of species other than *Bacillus* (see Materials and Methods). Genomic regions orthologous to putative TRGs are identified using the reciprocal tFASTy/BLASTp approach. Orthologous genomic regions are supported by synteny information by checking the conservation of genes flanking putative TRG. TRGs with reciprocal hits against the genomic region or lacking similarity against genomes other than *Bacillus* were included for analyses listed in the right (grayed) panel. Accordingly, the putative TRGs are queried in the reciprocal BLASTp/BLASTp and tFASTy/BLASTp searches for genus/species-level TRG orthologs/paralogs across *Bacillus* species. If a query putative TRG had ortholog(s)/paralog(s) that could not be classified as taxonomically restricted, the candidate TRG was classified as false-positive and removed from further analyses (for more information see Figure 2).

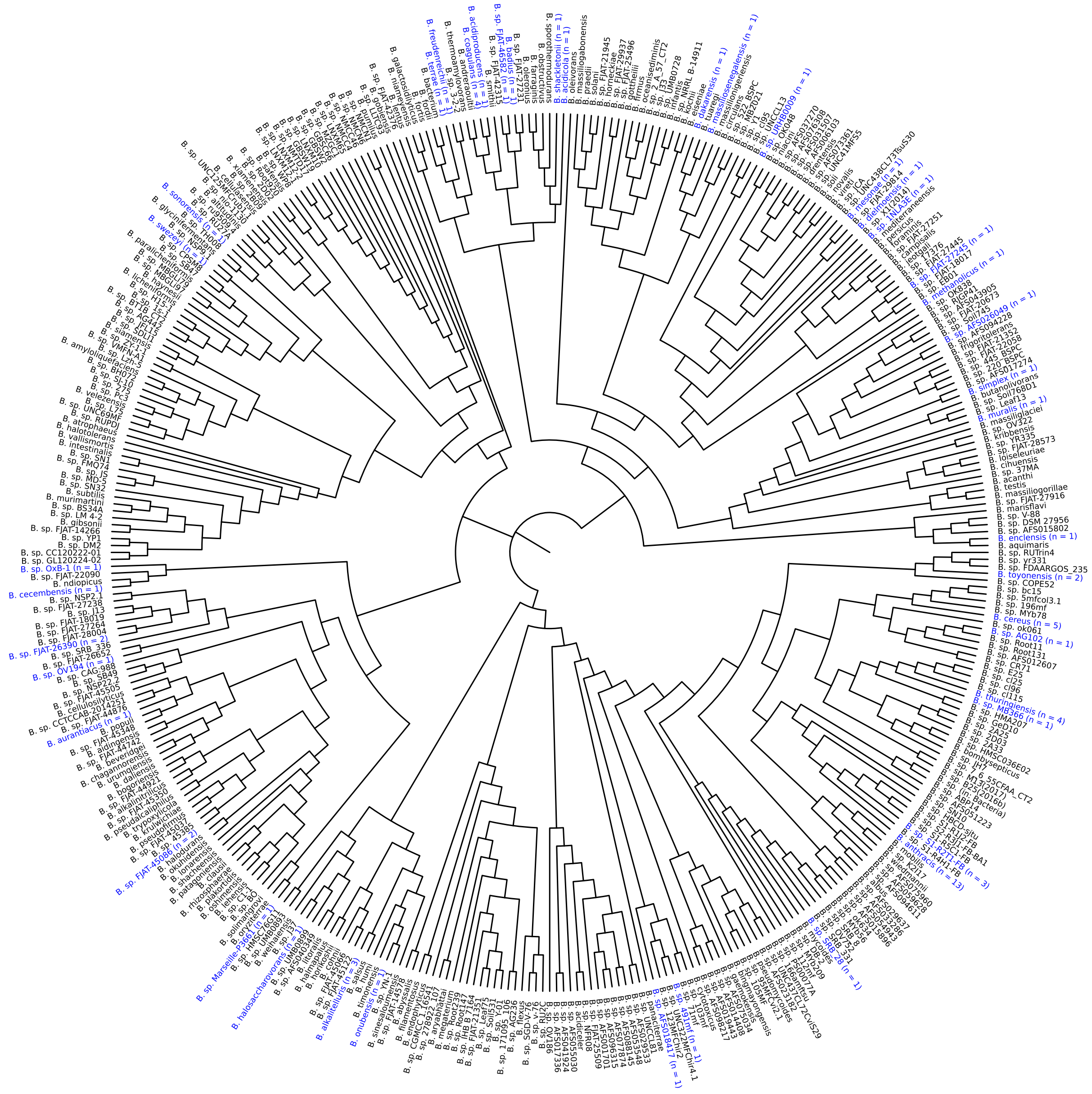

Figure S2. Whole-genome neighbor-joining phylogeny inferred from Mash distances of *Bacillus* genomes belonging to 396 species. The *Bacillus* genomes with at least one taxonomically restricted gene (TRG) are highlighted in blue, with the number of TRGs indicated in parentheses.

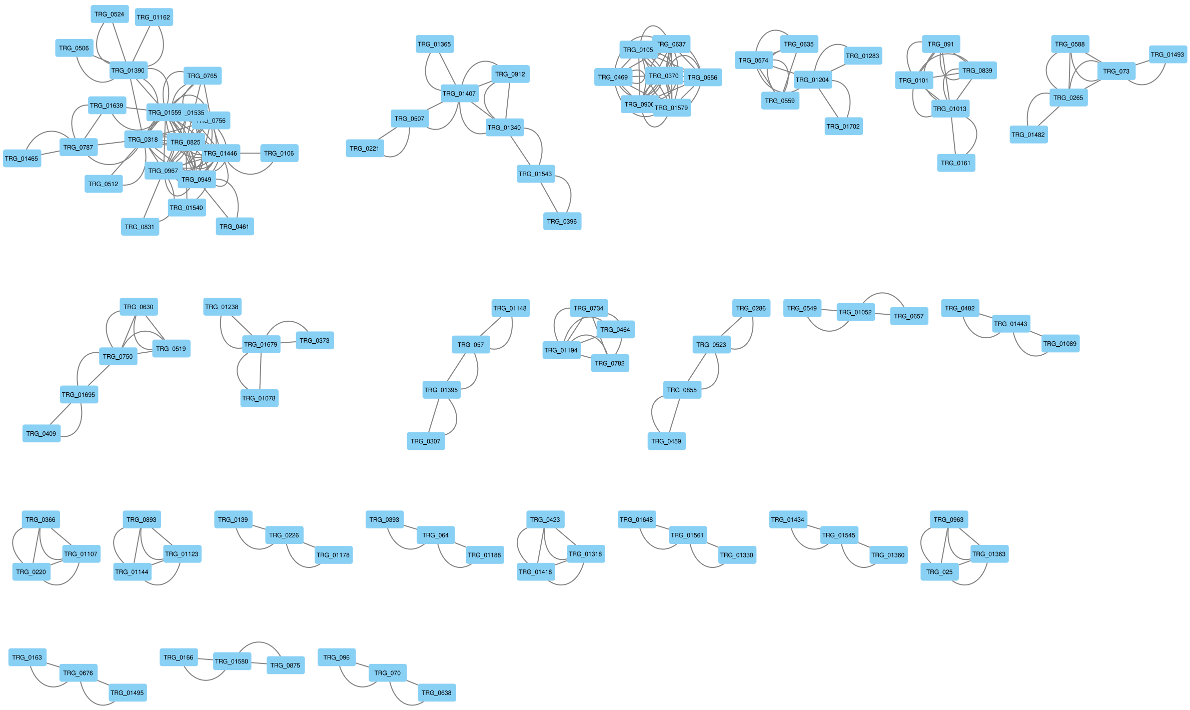

Figure S3. TR gene clusters with more than two members. Cytoscape graphic representation of cluster structures. Nodes depict TRG proteins and edges represent reciprocal best BLAST hits (two lines between a pair of sequences show the forward and backward BLAST search).

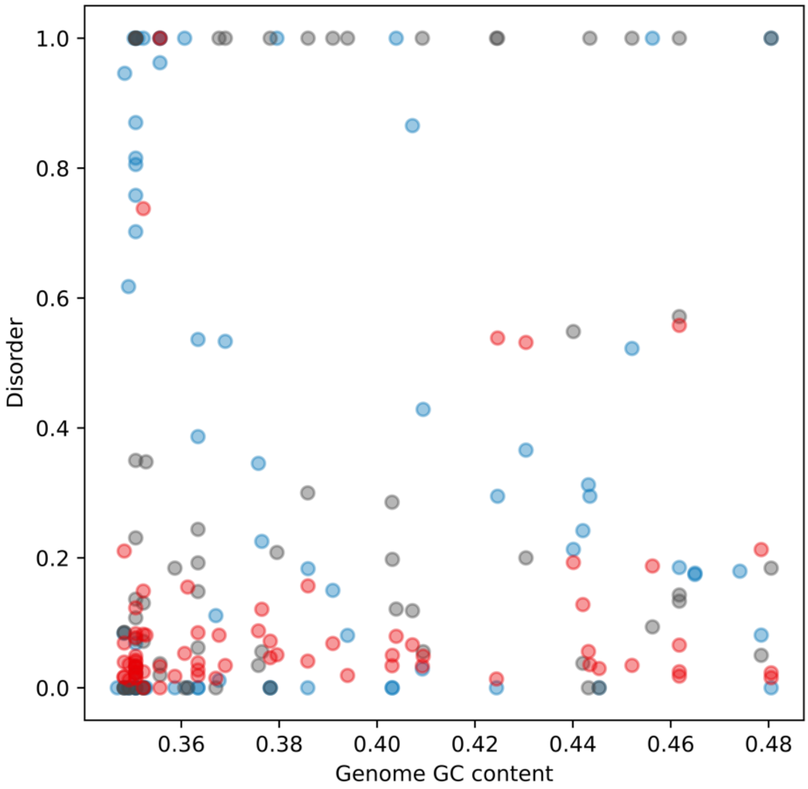

Figure S4. Relation between genome GC-content and the disorder score of species- level TRGs. TRGs (blue dots) are compared to nonannotated open reading frame (ORF) sequences (grey dots) and annotated non-TRG proteins (red dots).
